# Single-subject analysis of regional brain volumetric measures can be strongly influenced by the method for head size adjustment

**DOI:** 10.1007/s00234-022-02961-6

**Published:** 2022-04-25

**Authors:** Roland Opfer, Julia Krüger, Lothar Spies, Hagen H. Kitzler, Sven Schippling, Ralph Buchert

**Affiliations:** 1 Jung Diagnostics GmbH, Hamburg, Germany; 2 Institute of Diagnostic and Interventional Neuroradiology, University Hospital Carl Gustav Carus, Technische Universität Dresden, Dresden, Germany; 3 Center for Neuroscience Zurich (ZNZ), Federal Institute of Technology (ETH), Multimodal Imaging in Neuroimmunological Diseases (MINDS), University of Zurich, Zurich, Switzerland; 4 Department of Diagnostic and Interventional Radiology and Nuclear Medicine, University Medical Center Hamburg-Eppendorf, Martinistr. 52, 20246 Hamburg, Germany

**Keywords:** MRI, Total intracranial volume, Brain parenchyma, Thalamus, Hippocampus, Age

## Abstract

**Purpose:**

Total intracranial volume (TIV) is often a nuisance covariate in MRI-based brain volumetry. This study compared two TIV adjustment methods with respect to their impact on *z*-scores in single subject analyses of regional brain volume estimates.

**Methods:**

Brain parenchyma, hippocampus, thalamus, and TIV were segmented in a normal database comprising 5059 T1w images. Regional volume estimates were adjusted for TIV using the residual method or the proportion method. Age was taken into account by regression with both methods. TIV- and age-adjusted regional volumes were transformed to *z*-scores and then compared between the two adjustment methods. Their impact on the detection of thalamus atrophy was tested in 127 patients with multiple sclerosis.

**Results:**

The residual method removed the association with TIV in all regions. The proportion method resulted in a switch of the direction without relevant change of the strength of the association. The reduction of physiological between-subject variability was larger with the residual method than with the proportion method. The difference between *z*-scores obtained with the residual method versus the proportion method was strongly correlated with TIV. It was larger than one *z*-score point in 5% of the subjects. The area under the ROC curve of the TIV- and age-adjusted thalamus volume for identification of multiple sclerosis patients was larger with the residual method than with the proportion method (0.84 versus 0.79).

**Conclusion:**

The residual method should be preferred for TIV and age adjustments of T1w-MRI-based brain volume estimates in single subject analyses.

**Supplementary Information:**

The online version contains supplementary material available at 10.1007/s00234-022-02961-6.

## Introduction

MRI-based regional brain volume estimates are increasingly used to support diagnosis and monitoring of neurological and psychiatric diseases. For example, MRI-based hippocampus volume is used as a topographical biomarker to detect or exclude hippocampal degeneration in patients with suspected Alzheimer’s disease [1]. In multiple sclerosis (MS), thalamus atrophy detected by MRI is amongst the earliest signs of neurodegeneration and a strong predictor of disability and cognitive impairment [2].

For most regions-of-interest (ROI) in the brain, MRI-based volume estimates (ROIV) are associated with head size [3, 4]. Thus, head size can be a confounder of no interest (nuisance covariate) in between-subject comparisons [3]. Careful adjustment for head size is required in these cases not only to improve the statistical power to detect effects of interest, but also to avoid spurious effects and misinterpretation of actual effects [3, 5, 6]. When considering ROIV estimates across the entire adult age range, the total intracranial volume (TIV) derived from the same T1w-MRI as ROIV is often used to account for head size [3, 7].

Commonly used methods for TIV adjustment of ROIV estimates include the residual method and the proportion method [5, 7–9]. In the residual method, ROIV is regressed on TIV in a sufficiently large, independent group of normal control subjects (normal database). The resulting regression model is used to compute the residuals of ROIV relative to the model in the subjects of interest (usually not included in the normal database). The residuals quantitatively characterize the extent to which each individual subject’s ROIV deviates from that predicted for normal subjects with the same TIV. With the proportion method, the ROIV is scaled to the individual TIV to obtain the ROIV-to-TIV ratio, denoted as ROIF in the following (“F” for fraction).

The major advantage of the proportion method is that it is intuitive and straightforward to implement. In particular, it does not require an independent database of normal control subjects. A disadvantage of the proportion method is that it often does not remove the association of ROIV with TIV, whereas the residual method does [5, 7, 9–11].

A further advantage of the residual method is that it easily allows adjustment for other nuisance covariates such as age simultaneously with TIV, simply by adding further terms to the regression model. When using the proportion method for TIV adjustment, adjustment for other nuisance covariates can be achieved by computing residuals of ROIF with respect to a regression model of ROIF in the normal database with the other nuisance variables as explanatory variables. However, often, there is no clear rationale for treating TIV separately and differently from the other nuisance variables. Furthermore, curvilinear (allometric) relationships [4] between regional brain volumes and TIV are easily modelled with the residual method by including logarithmic, quadratic, or higher order polynomial terms in the regression model. The same is true for interaction between different nuisance covariates, e.g., TIV and age, on the regional brain volume of interest, by adding an interaction term to the regression model. Finally, one of the major assumptions of the residual method to be valid, namely that in the normal database the residuals are normally distributed, is easily checked for quality control. There is no analogous quality check of proportions.

The impact of the TIV adjustment method on regional brain volumetry has been studied before. However, previous studies entirely focused on group level analyses to characterize disease-specific effects [10, 12, 13], or the impact of age and gender on brain volumetric measures [4, 8, 14–19], or associations between brain volumetric measures and cognitive performance [11]. The aim of the present study was to evaluate the impact of the TIV adjustment method on *z*-scores used to support the interpretation of regional brain volume estimates in single subject analyses of individual patients. T1w-MRI-based volume estimates of brain parenchyma (large volume), thalamus (intermediate volume), and hippocampus (small volume) were considered as clinically relevant examples. The regional brain volumes were adjusted for age in addition to TIV, because longitudinal and cross-sectional studies clearly demonstrated age-related shrinkage of the brain independent of the TIV [20, 21].

## Materials and methods

### Normal database of T1w-MRI scans

The normal database comprised 5059 clinical 3D gradient echo T1w-MR images of the brain from 5059 different patients referred to MR imaging for unspecific symptoms (headache, dizziness). The age of the patients ranged between 20 and 90 years (mean 49.6 ± 16.7 years, the age distribution is shown in Online Supplementary Fig. 1); 57.6% were females (sex was not known in 540 patients). None of the patients had a history of or currently ongoing neurological or psychiatric disease at the time of MR imaging. All images were free of abnormalities beyond those expected for the patients’ age based on visual inspection by the local radiologists. Clinical follow-up after MRI was not available. The images had been acquired with 160 different MRI scanners from three different manufacturers (Siemens/Philips/GE: *n* = 109/37/14). Most of the scanners operated at 1.5 T field strength (*n* = 116), the remaining scanners at 3 T (*n* = 44). The MR images had been acquired with scanner-specific sequences as recommended for brain volumetric analyses by the scanner manufacturers. The slice thickness ranged between 0.9 and 1.3 mm.

The MR images of the normal database had been acquired in clinical routine and were transferred to jung diagnostics GmbH under the terms and conditions of the European general data protection regulation for remote image analysis. Subsequently, the data had been anonymized. The need for written informed consent for the retrospective use of the anonymized data in the present study was waived by the ethics review board of the general medical council of the state of Hamburg, Germany (reference number 2021–300,047-WF).

### MS patient dataset

T1w-MR images of 127 MS patients were included retrospectively to compare the two TIV adjustment methods with respect to their impact on the sensitivity to detect thalamus atrophy in MS.

Thirty-three of the MS patients had been enrolled in a clinical study at the Institute of Diagnostic and Interventional Neuroradiology of the University Hospital Carl Gustav Carus, Dresden, Germany (age 42.2 ± 10.1 years, Expanded Disability Status Scale 2.7 ± 1.6, disease duration 5.2 ± 4.8 years). T1w-MR images had been acquired with a 3.0 T Siemens Verio scanner. The remaining 94 MS patients had participated in an observational study at the University Hospital of Zurich, Switzerland (age 37.3 ± 8.9 years, Expanded Disability Status Scale 1.3 ± 1.3, disease duration 2.7 ± 4.5 years). The T1w-MR images had been acquired with a 3.0 T Philips Ingenia scanner. Both studies used a 3D gradient echo T1w-MR sequence.

Both studies had been approved by the local ethics committee. All patients had given written informed consent. This included the retrospective use of the data for the present study. All procedures were in accordance with the 2013 Helsinki declaration.

The MRI data of the MS subsamples have been used previously in a study on age-dependent cutoffs for the detection of pathological deep gray matter volume loss using Jacobian integration [22].

### Regional volumetry

A custom three-dimensional convolutional neural network with U-net architecture [23] recently introduced for the segmentation of pathological lesions in T2w-MRI [24, 25] was used to segment the whole brain parenchyma (BP, = gray and white matter), the total intracranial volume (TIV, = BP + inner and outer cerebrospinal fluid), the hippocampi (HIPP), and the thalamus (THAL) in each T1w-MRI. Training and validation of the 3D-CNN for segmentation of these brain structures with respect to stability and accuracy will be described elsewhere. The segmentation results were used to compute the corresponding volumes in ml in each T1w-MRI (TIV, BPV, THALV, HIPPV). THALV and HIPPV comprised thalamus and hippocampus in both hemispheres.

### TIV and age adjustments with the residual method

For the residual method [17–19, 26], the physiological relationship of the regional volume ROIV with TIV and age was modelled in the normal database by

1 $$\mathrm{ROIV}=a\times {\mathrm{TIV}}^{2}+b \times {\mathrm{age}}^{2}+c\times \mathrm{TIV}\times \mathrm{age}+d\times TIV+e\times \mathrm{age}+f.$$

More precisely, the regression coefficients *a*, *b*, *c*, *d*, *e*, and *f* were determined by minimizing the sum of squared differences between the individual ROIV estimates in the normal database and the model according to Eq. 1. Then, the residual of an individual ROIV estimate with respect to the model, denoted resROIV, was computed as

2 $$\mathrm{resROIV}=\mathrm{ ROIV}-\left(a\times {\mathrm{TIV}}^{2}+b \times {\mathrm{age}}^{2}+c\times \mathrm{TIV}\times \mathrm{age}+d\times \mathrm{TIV}+e\times \mathrm{age}+f\right).$$

### TIV and age adjustments with the proportion method

First, the individual ROIV was divided by the individual TIV to obtain ROIF, the ROIV-to-TIV fraction [27–29]. Then, adjustment for age was performed by computing residuals of ROIF with respect to the following model

3 $$\mathrm{ROIF}=r\times {\mathrm{age}}^{2}+s\times \mathrm{age}+t$$

The regression coefficients *r*, *s*, and *t* were determined by minimizing the sum of squared differences between the individual ROIF estimates in the normal database and this model. The residual of individual ROIF estimates with respect to this model was computed as

4 $$\mathrm{resROIF}=\mathrm{ ROIF}-\left(r\times {\mathrm{age}}^{2}+s\times \mathrm{ age}+t\right).$$

### Outliers

Regression can be affected by outliers. In order to account for this, an iterative two-step approach was used for the regression modelling. After the first regression, scans with residual < lower quartile − 1.5 × inter-quartile-range or residual > upper quartile + 1.5 × inter-quartile range were considered outliers and were excluded from the second (and final) regression. The identification of outliers was performed separately for the residual method and for the proportion method and separately for each ROI.

### Statistical analysis

The coefficient of variance (CoV[%] = 100 × standard deviation/mean) in the normal database was used to characterize physiological between-subject variability of raw and adjusted volume measures. The CoV of residuals was computed using the mean value of the corresponding raw measure as reference (residuals have zero mean).

Adjusted volumes were tested for (residual) correlation with TIV and age by bivariate correlation analysis using Pearson’s method.

*Z*-scores of the residuals were used for standardized characterization of the deviation of TIV- and age-adjusted ROIV from the norm, independent from the adjustment method [30]. Residuals were transformed to *z*-scores by scaling them to the standard deviation of the residuals in the normal database, that is,

5 $$z-\mathrm{resROIV}=\mathrm{resROIV}/\mathrm{standard}\;\mathrm{deviation}\;\mathrm{of}\;\mathrm{resROIV}$$

and

6 $$z-\mathrm{resROIF}=\mathrm{resROIF}/\mathrm{standard}\;\mathrm{deviation}\;\mathrm{of}\;\mathrm{resROIF}.$$

The individual difference between *z*-resROIF (obtained with the proportion method) and *z*-resROIV (obtained with the residual method), that is,

7 $$z-\mathrm{diff }= z-\mathrm{resROIF}- z-\mathrm{resROIV}$$

was used to assess the impact of the adjustment method on the interpretation of regional brain volume estimates in individual subjects. The *z*-score difference (*z*-diff) was tested for possible association with TIV and age.

The impact of TIV adjustment on the power to detect MS-associated thalamus atrophy was assessed by comparing the receiver operating characteristic (ROC) curves to separate the MS patients from the control subjects in the normal database between *z*-resTHALV and *z*-resTHALF.

## Results

**Fig. 1:**
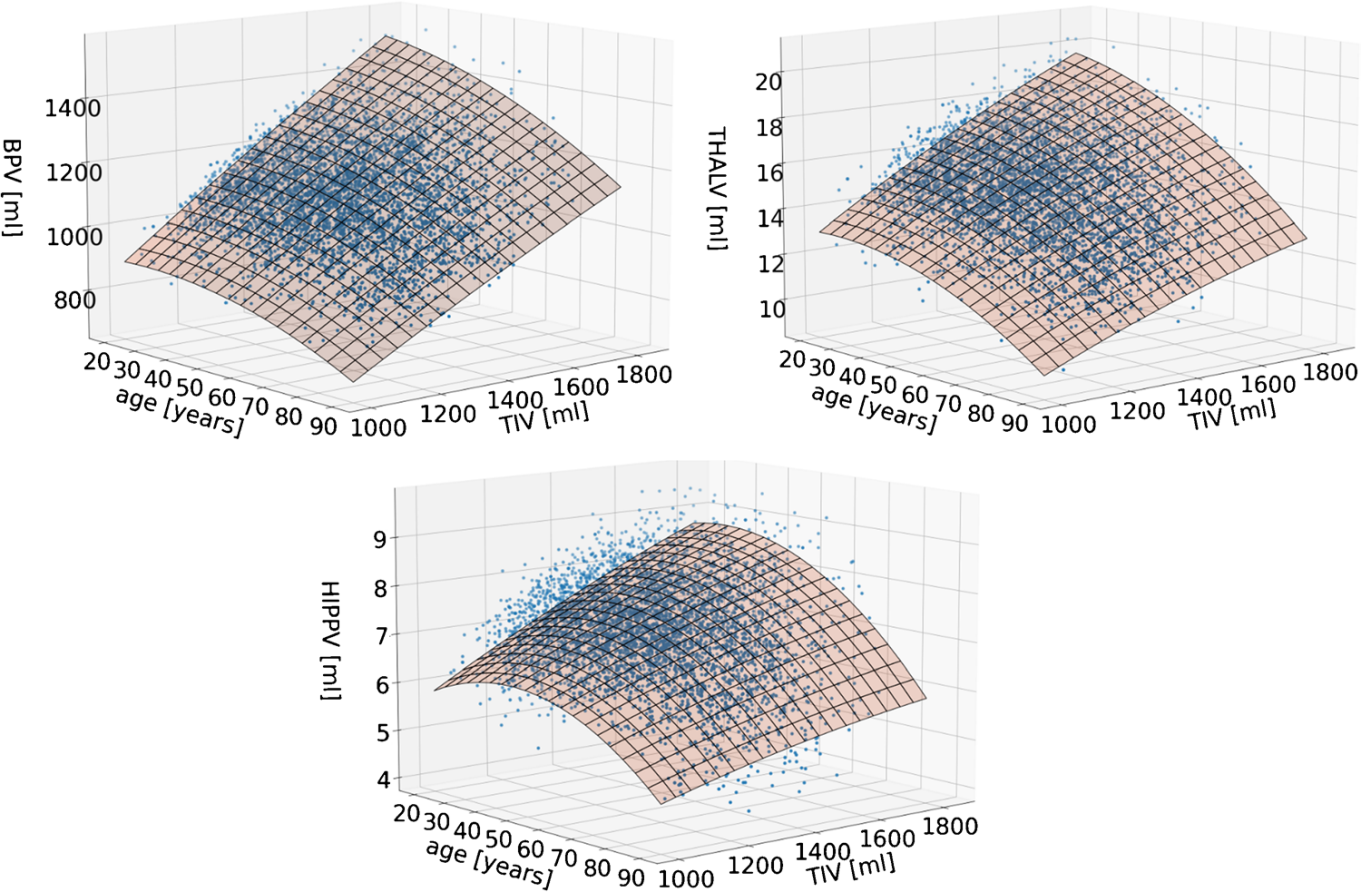
Final regression models of the relationship of ROIV with TIV and age obtained for the residual method (top left: BPV, top right: THALV, bottom: HIPPV)

**Fig. 2:**
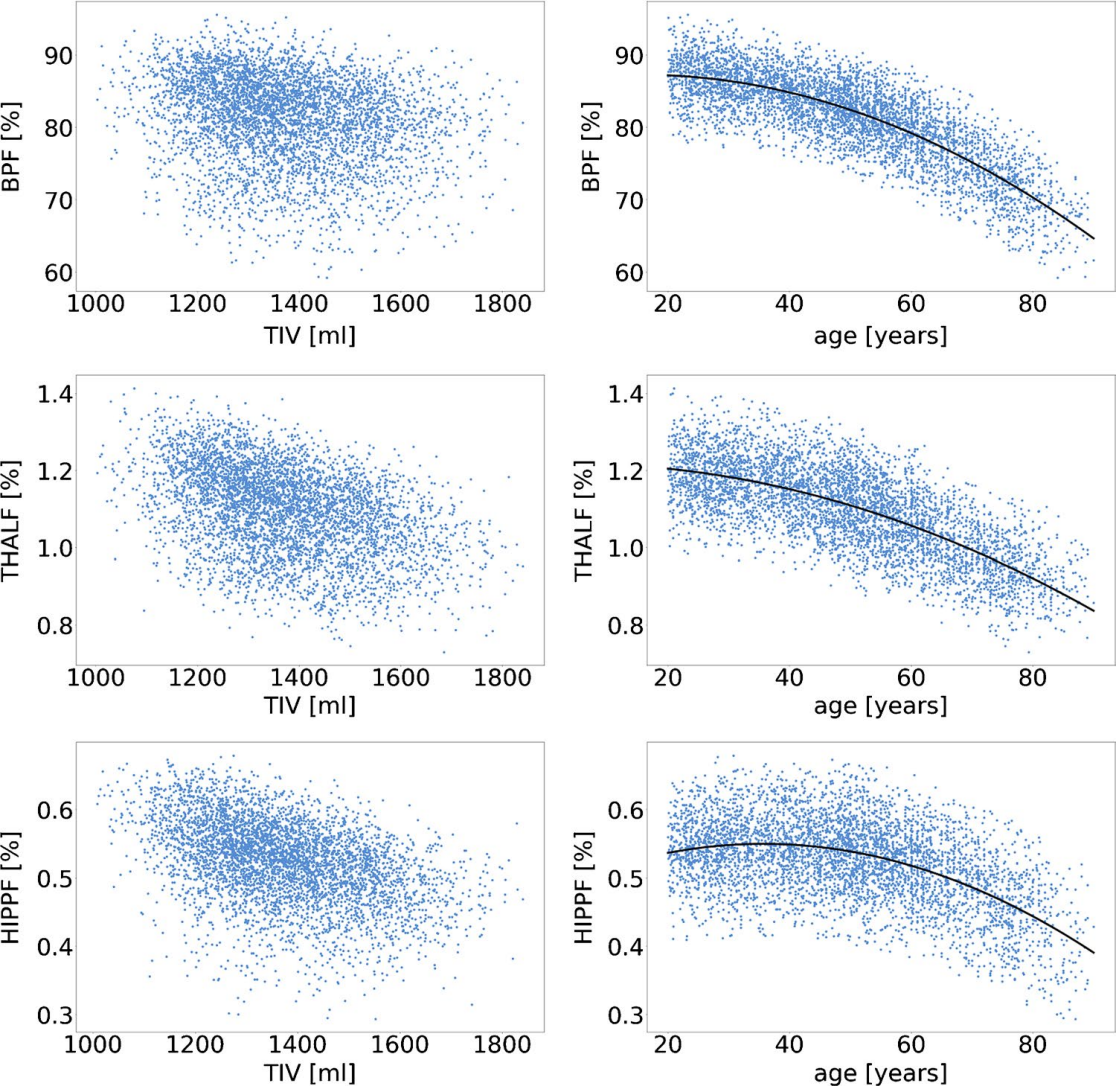
Scatter plots of ROIF versus TIV (left) and the final regression models of the relationship of ROIF with age for the proportion method (right) (top: BP, middle: THAL, bottom: HIPP)

The number of outliers of the residual after the first iteration of regression modelling in the normal database was 79 (BP), 71 (THAL), and 104 (HIPP) for the residual method and 64, 52, and 73 for the proportion method. Figure 1 shows the final regression models obtained with the residual method. Scatter plots of ROIF versus TIV are shown in Fig. 2, together with the final regression models of ROIF versus age. All terms except the ${\mathrm{TIV}}^{2}$ term (coefficient $a$) of the final regression model of the residual method contributed significantly.

The distribution of the residuals in the normal database (without the outliers) was Gaussian to good approximation in all cases (Online Supplementary Fig. 2).

Scaling the regional volume (ROIV) to the TIV to obtain the ROIV-to-TIV fraction (ROIF) for the proportion method resulted in a considerable decrease of the CoV for BP (from 10.54 to 6.70%; Table 1), a small decrease for THAL (from 11.17 to 10.41%), and a small *in*crease of the CoV for HIPP (from 11.26 to 11.41%). Adjustment for age in the proportion method resulted in a decrease of the CoV for all ROIs (BP: from 6.70 to 4.04%, THAL: from 10.41 to 7.14%, HIPP: from 11.41 to 9.65%; Table 1). When comparing TIV- and age-adjusted measures between the residual method and the proportion method, the CoV was lower with the residual method for all ROIs (BP: 3.68% versus 4.04%, THAL: 5.95% versus 7.14%, HIPP: 8.16% versus 9.65%; Table 1).

**Table 1:** Mean value, standard deviation (*SD*), and coefficient of variance (CoV[%] = 100 × *SD*/mean) of adjusted and non-adjusted volume estimates of brain parenchyma (BP), bilateral thalamus (THAL), and bilateral hippocampus (HIPP) in the normal database. The last two columns give the Pearson correlation coefficients with TIV and age. Statistically significant (*P* < .0001) correlations are marked with an asterisk

Residual method						
ROI	Adjustment	Volume (ROIV)			Correlation with	
		Mean [ml]	*SD* [ml]	CoV [%]	TIV	age
BP (*n* = 4980)	No adjustment	1112.66	117.28	10.54	0.79*	− 0.40*
	After adjustment	0.00	40.96	3.68	0.00	− 0.00
THAL (*n* = 4988)	No adjustment	14.97	1.67	11.17	0.52*	− 0.57*
	After adjustment	0.00	0.89	5.95	0.00	0.00
HIPP (*n* = 4955)	No adjustment	7.16	0.81	11.26	0.42*	− 0.38*
	After adjustment	0.00	0.58	8.16	− 0.00	− 0.00
Proportion method						
ROI	Adjustment	Proportion of TIV (ROIF)			Correlation with	
		Mean [%]	*SD* [%]	CoV [%]	TIV	Age
BP (*n* = 4995)	No adjustment	81.3	5.45	6.70	− 0.31*	− 0.76*
	After adjustment	0.00	3.28	4.04	− 0.39*	− 0.00
THAL (*n* = 5007)	No adjustment	1.10	0.11	10.41	− 0.42*	− 0.70*
	After adjustment	0.00	0.08	7.14	− 0.52*	0.00
HIPP (*n* = 4986)	No adjustment	0.52	0.06	11.41	− 0.45*	− 0.45*
	After adjustment	0.00	0.05	9.65	− 0.52*	− 0.00

Raw ROIV was positively correlated with TIV and negatively with age in all ROIs (Table 1). The residual method removed the correlation with TIV and age in all ROIs, whereas the proportion method removed only the correlation with age (Table 1). The sign of the correlation with TIV was switched by the proportion method in all ROIs (Table 1).

**Fig. 3:**
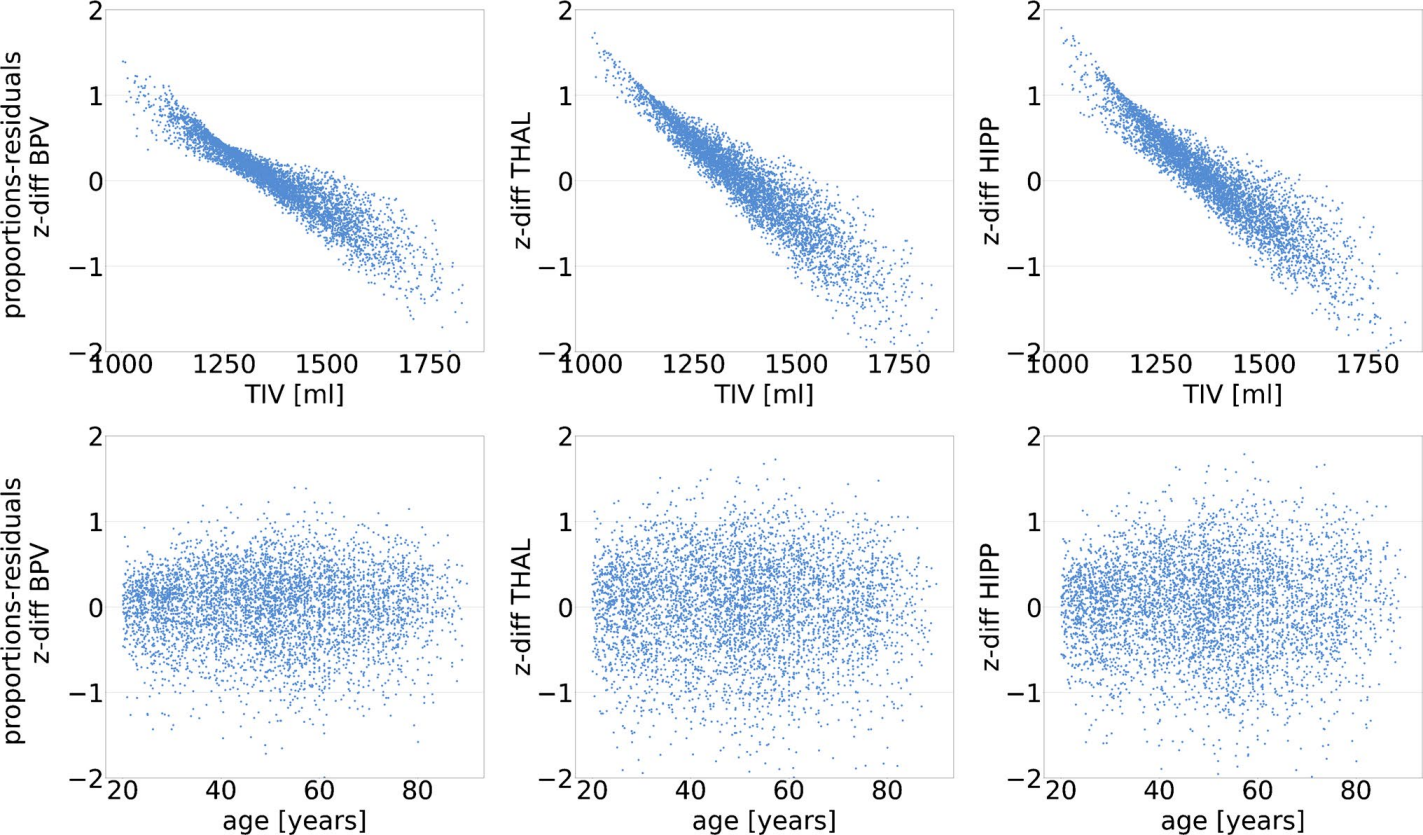
Scatter plots of *z*-diff versus TIV (top) and versus age (bottom) (left: BP, middle: THAL, right: HIPP)

The distribution of *z*-diff, the individual difference of the regional brain volume *z*-scores with the proportion method minus the residual method, in the normal database is shown in Online Supplementary Fig. 3. Mean, 95th percentile, and maximum of the absolute value of *z*-diff are given in Table 2. The maximum *z*-score difference was larger or equal to two *z*-value points in all ROIs. Scatter plots of *z*-diff versus TIV and versus age are shown in Fig. 3. There was a strong correlation between *z*-diff and TIV in all ROIs (*r* <  − 0.92, *p* < 0.001). Relative to the residual method, the proportion method resulted in overestimation of individual *z*-scores at small TIV and in underestimation of *z*-scores at large TIV. *z*-diff was not associated with age (Fig. 3).

**Table 2:** Absolute difference of regional brain volume *z*-scores with the proportion method minus the residual method (abs(*z*-diff)) in the normal database

ROI	Mean	95th percentile	Max
BPV	0.34	0.86	2.00
THALV	0.46	1.11	2.05
HIPPV	0.44	1.09	2.20

The ROC curves of *z*-resTHALV and *z*-resTHALF for the discrimination of MS patients from the control subjects in the normal database are shown in Online Supplementary Fig. 4. The area under the ROC curve was 0.84 for *z*-resTHALV and 0.79 for *z*-resTHALF.

## Discussion

The residual method was superior to the proportion method with respect to reduction of physiological between-subject variability and with respect to removing the association with TIV of the considered regional brain volumes. The first suggests improved statistical power with the residual method, which was supported by a higher area under the ROC curve with the residual method when testing the thalamus volume for the discrimination of MS patients from control subjects (Online Supplementary Fig. 4). The latter suggests an increased risk of systematic misinterpretation of MRI-based volume estimates with the proportion method, depending on the subject’s TIV.

The mean difference of the *z*-scores from the two adjustment methods ranged between 0.34 and 0.46 *z*-score points, depending on the ROI (Table 2), which might appear rather small. However, the *z*-score difference was larger than one *z*-score point in about 5% of the 5059 scans in each ROI (Table 2). The maximum *z*-score difference observed in this sample was about two *z*-score points in each ROI (Table 2). Very similar findings were obtained for hippocampus volumetry using two alternative segmentation methods suggesting that the findings are not specific for segmentation with the custom convolutional neuronal network used in this study but hold more generally (section “Impact of the segmentation method” in the Online Supplementary material). Given that typical cutoffs used for the detection of regional brain atrophy range between − 1.5 and − 2.5 on the *z*-score, a systematic error of one *z*-score point or more in about 5% of the subjects questions the use of the proportion method in single subject analyses of MRI-based brain volumetry in clinical routine, although it is still widely used [31, 32].

The findings of the present study are in line with previous studies demonstrating that the proportion method often does not remove the association of ROIV estimates with TIV [5, 7, 9–11]. The proportion method implicitly assumes that in healthy subjects, ROIV is proportional to the TIV, that is, ROIV = slope × TIV. In this case, the fraction ROIF of ROIV relative to the TIV is constant (independent of TIV): ROIF = ROIV/TIV = slope. The fact that the proportion method did not remove the association between ROIV and TIV in the present study suggests that the assumption of proportionality between ROIV and TIV was violated in the patient data by a non-zero intercept: ROIV = slope × TIV + intercept [4]. In this case, it is ROIF = ROIV/TIV = slope + intercept/TIV. Thus, a positive intercept results in an inverse association between ROIF and TIV (smaller ROIF at larger TIV) as observed in this study (Fig. 2) [11, 12]. The sign flip of the correlation with TIV was observed for all ROIs. For the HIPP, the proportion method even caused a slight increase of the correlation strength (correlation coefficient from 0.42 to − 0.52; Table 1). Thus, if the goal is to eliminate the impact of variable TIV on regional brain volume measures, the residual method is strongly preferred over the proportion method [11].

A secondary finding of the present study was the statistical significance of the TIV × age interaction term in the regression model used for the residual method (Eq. 1), indicating that the relationship between ROIV and TIV depends on age in all considered ROIs. Evaluating the final regression model of the residual method at different fixed ages demonstrated a steeper relationship between ROIV and TIV in younger subjects compared to older subjects (Online Supplementary Fig. 5). TIV × age interaction cannot be taken into account with the proportion method. The same is true for the residual method when applied stepwise, that is, when TIV is regressed out first and the resulting residuals are subsequently regressed over age [21, 33]. This stepwise approach is expected to result in overestimation of *z*-scores in young subjects (reduced sensitivity to detect volume loss) and in underestimation of *z*-scores in old subjects (increased rate of false positive findings).

Another secondary finding was the statistical significance of the quadratic age term in the regression model used for the residual method (Eq. 1). This indicates that a simple linear model is not sufficient to adequately describe the age dependency of regional brain volume over the entire range of adult age, in line with previous studies [22, 33]. Non-linear age effects can be taken into account with both the residuals and the proportion method. In this study, a quadratic age term was included in the regression model of both methods in order to avoid bias that might have been caused by including this term in only one method (Eqs. 1 and 3).

Analysis of covariance is also widely used for TIV adjustment with ROIV as dependent variable and TIV and other nuisance covariates as predictors together with the factors of primary interest such as disease status for example [7, 34]. However, analysis of covariance is designed for the comparison of two or more groups and, therefore, was not considered in this study on the impact of TIV adjustment on single-subject analyses.

Matching subjects with respect to all relevant nuisance variables might be considered the standard-of-truth to account for them. This is feasible in group comparisons [15, 18]. However, in single-subject analysis, this would require a huge normal database that can be stratified with respect to TIV and age such that each combination of TIV and age encountered in clinical routine is covered with adequate sample size. Such databases are currently not available at most sites.

Several parameters have been proposed to account for between-subject variability of head size in studies on regional brain volumes in addition to the TIV including body height, head circumference, and total brain volume (excluding ventricles) [7]. Which parameter is most appropriate depends on the question of interest [7]. When considering regional brain volumes across the entire adult age range and/or to support the diagnosis of neurodegenerative diseases, the TIV usually is preferred, because it is rather independent of age and atrophy and, therefore, can be considered an index of maximum brain volume across the individual’s entire adult life span [3, 7, 35, 36]. This was the rationale for using the TIV for head size adjustment in the present study. Furthermore, there are numerous software tools for automatic estimation of the TIV from the same high-resolution T1w-MRI of the brain (as used for regional volumetry) with high accuracy and precision. Thus, reliable TIV estimates are easily available to account for head size with only moderate amplification of noise (measurement error).

Hyperostosis frontalis interna, characterized by bony nodules situated on the inner lamina of the frontal (and parietal) bone, causes underestimation of the head size by the TIV. Hyperostosis frontalis interna is rather frequent in post-menopausal females but rarely found in males [37]. Repeat analyses performed separately in females and males suggested that the potential impact of limitations of TIV estimates associated with hyperostosis frontalis interna on the primary findings of the present study was small (section “Potential impact of hyperostosis frontalis interna” in the Online Supplementary material).

A limitation of the present study is that the interpretation of individual terms in the regression models might be limited by multicollinearity between the terms. However, this does not affect the utility of the full regression models to reduce between-subject variability associated with TIV and age.

## Conclusion

The residual method should be preferred over the proportion method for TIV and age adjustments of T1w-MRI-based brain volume estimates for the detection or exclusion of regional brain atrophy in single subjects in clinical routine.

## Supplementary Information

Below is the link to the electronic supplementary material.

Supplementary file1 (PDF 1029 kb)

## Abbreviations

BP/BPV/BPF: Brain parenchyma/BP volume (ml)/fraction of BPV relative to the TIV (%)
CoV: Coefficient of variance
HIPP/HIPPV/HIPPF: Hippocampus/bilateral HIPP volume (ml)/fraction of HIPPV relative to the TIV (%)
THAL/THALV/THALF: Thalamus/bilateral THAL volume (ml)/fraction of THALV relative to the TIV (%)
ROC: Receiver operating characteristic
ROI/ROIV/ROIF: Region-of-interest/ROI volume (ml)/fraction of ROIV relative to the TIV (%)
*SD*: Standard deviation
TIV: Total intracranial volume

## Declarations

The MR images of the normal database had been transferred to jung diagnostics GmbH under the terms and conditions of the European general data protection regulation for remote image analysis. Subsequently, the data had been anonymized. The need for written informed consent for the retrospective use of the anonymized data in the present study was waived by the ethics review board of the general medical council of the state of Hamburg, Germany (reference number 2021–300047-WF).

The MS patients had been enrolled in clinical studies at the Institute of Diagnostic and Interventional Neuroradiology of the University Hospital Carl Gustav Carus, Dresden, Germany, and at the University Hospital of Zurich, Switzerland. Both studies had been approved by the local ethics committee. All patients had given written informed consent. This included the retrospective use of the data for the present study.

All procedures were in accordance with the 2013 Helsinki declaration.
