# Supplementary material for: Single-subject analysis of regional brain volumetric measures can be strongly influenced by the method for head size adjustment
Source: Neuroradiology. 2022 Apr 25;64(10):2001–9. doi: 10.1007/s00234-022-02961-6 (PMC9474386; doi:10.1007/s00234-022-02961-6)
Supplement: Supplementary file 1 — Supplementary file1 (PDF 1029 kb) [file 234_2022_2961_MOESM1_ESM.pdf]

Supplementary Information

Title: Single subject analysis of regional brain volumetric measures can be strongly influenced by the method for head size adjustment

Journal: Neuroradiology

Authors: <blinded for review>

Affiliations: <blinded for review>

Corresponding author: <blinded for review>

Impact of the segmentation method

A custom three-dimensional convolutional neural network (3D-CNN) with U-net architecture [1] recently introduced for the segmentation of pathological lesions in T2w-MRI [2, 3] was used for the segmentation of the considered brain structures in T1w-MRI. Training and validation of the 3D-CNN for segmentation of these brain structures with respect to stability and accuracy will be described elsewhere.

To test whether the findings reported in the manuscript are specific for segmentation with this 3D-CNN, two alternative methods were used for hippocampus segmentation in the 5,059 T1w-MRI of the normal database: the recently introduced 2D-U-net-CNN implemented in the FastSurfer pipeline (https://github.com/Deep-MI/FastSurfer) and the FIRST module of the FSL Software (version 6.0.2; http://fsl.fmrib.ox.ac.uk/fsl). All analyses described in the manuscript were repeated for the FastSurfer and FSL hippocampus volume estimates in the normal database.

The following figure shows the scatter plots of the individual z-score difference (z-diff) between z-resHIPPF (obtained with the proportions method) and z-resHIPPV (obtained with the residuals method) versus TIV (top) and age (bottom) for hippocampus volume estimates obtained with FastSurfer (middle) and FSL (right). The corresponding scatter plots for hippocampus volume estimates obtained with the custom 3D-CNN are shown for comparison (left; same scatter plots as in the right column of Fig. 3 in the manuscript).

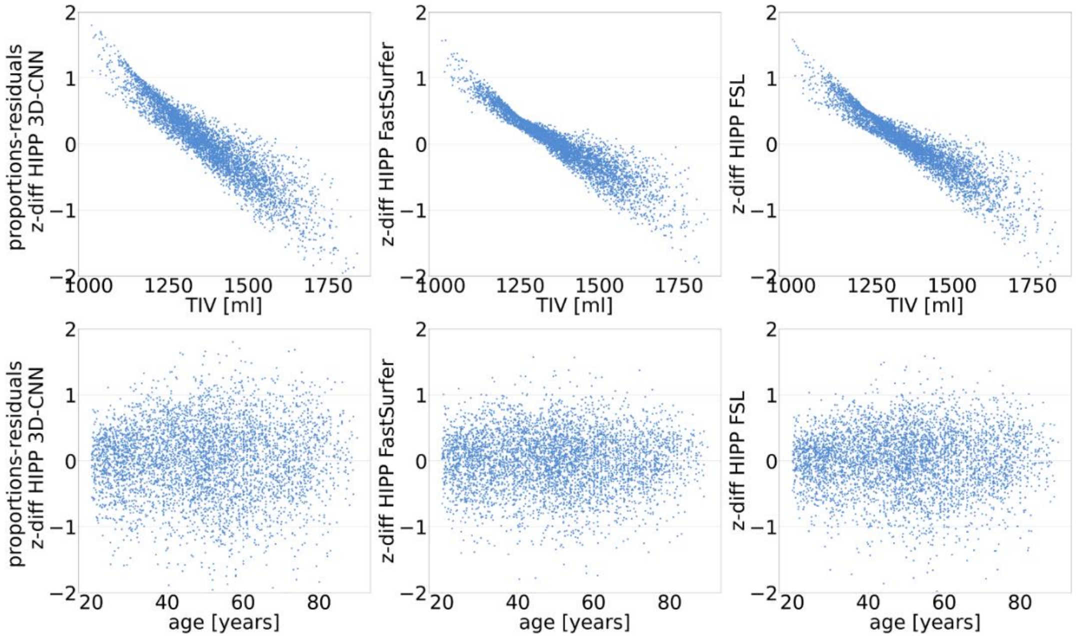

The scatter plots are very similar across the three different segmentation methods suggesting that the primary findings reported in the manuscript are independent of the segmentation method.

Potential impact of hyperostosis frontalis interna

Hyperostosis frontalis interna is characterized by bony nodules situated on the inner lamina of the frontal (and parietal) bone that result in underestimation of the head size by the TIV. Hyperostosis frontalis interna is rather frequent in post-menopausal females but rarely found in males [4].

To test for a potential impact of hyperostosis frontalis interna on the primary findings of the present study, the analyses of the bilateral hippocampus volume (from segmentation with the custom 3D-CNN) described in the manuscript were performed separately for females and males, including the regression analyses according to Eqs (1) and (3). Subjects with unknown sex were excluded (540 of 5,059).

The following figure shows the scatter plots of the individual z-score difference (z-diff) between z-resHIPPF (obtained with the proportions method) and z-resHIPPV (obtained with the residuals method) versus TIV (top) and age (bottom) in females (middle) and males (right). The scatter plots including both females and males are shown for comparison (left).

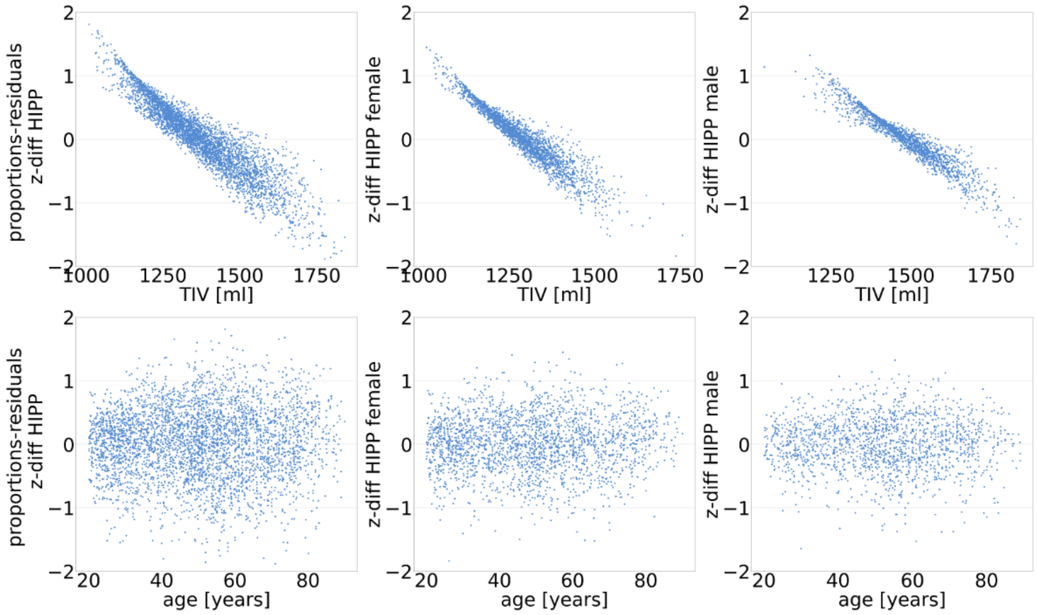

The scatter plots are similar for both genders suggesting that the potential impact of limitations of TIV estimates associated with hyperostosis frontalis interna on the primary findings of the present study is small.

References

1. Ronneberger O, Fischer P, Brox T (2015) U-net: Convolutional networks for biomedical image segmentation. In: International Conference on Medical image computing and computer-assisted intervention. Springer, pp 234-241

2. Krüger J, Opfer R, Gessert N, Ostwaldt AC, Manogaran P, Kitzler HH, Schlaefer A, Schippling S (2020) Fully automated longitudinal segmentation of new or enlarged multiple sclerosis lesions using 3D convolutional neural networks. NeuroImage Clinical 28:102445. https://doi.org/10.1016/j.nicl.2020.102445

3. Krüger J, Ostwaldt AC, Spies L, Geisler B, Schlaefer A, Kitzler HH, Schippling S, Opfer R (2021) Infratentorial lesions in multiple sclerosis patients: intra- and inter-rater variability in comparison to a fully automated segmentation using 3D convolutional neural networks. Eur Radiolhttps://doi.org/10.1007/s00330-021-08329-3

4. Raikos A, Paraskevas GK, Yusuf F, Kordali P, Meditskou S, Al-Haj A, Brand-Saberi B (2011) Etiopathogenesis of hyperostosis frontalis interna: a mystery still. Ann Anat 193:453-458. https://doi.org/10.1016/j.aanat.2011.05.004

Supplementary Figures

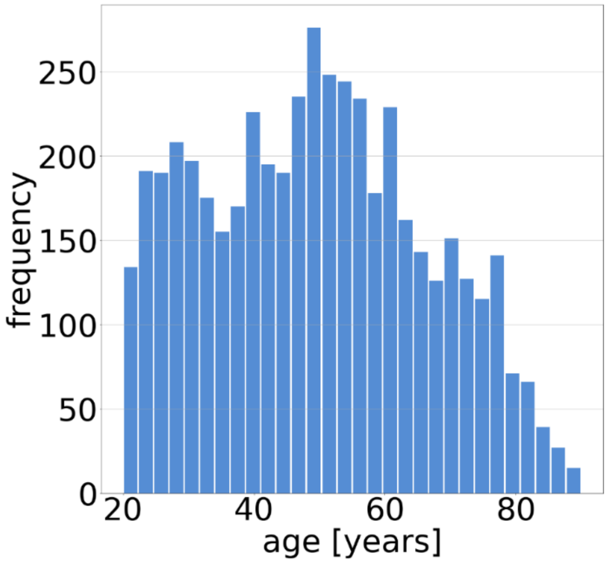

Supplementary Fig. 1 Age distribution of the 5,059 patients in the normal database

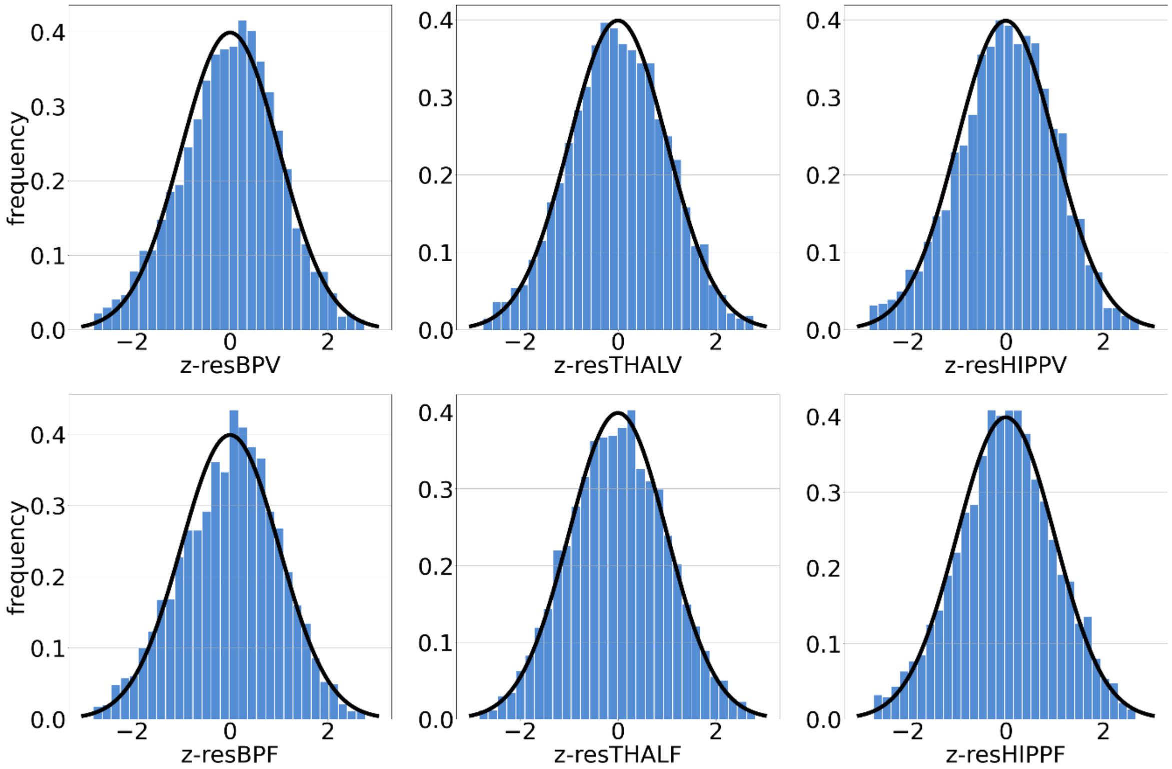

Supplementary Fig. 2 Distribution of the z-scores in the normal database after TIV- and age-adjustment with the residuals method (top) or the proportions method (bottom) (left: BP, middle: THAL, right: HIPP). The continuous line indicates the fit of the observed distributions by a gaussian distribution function

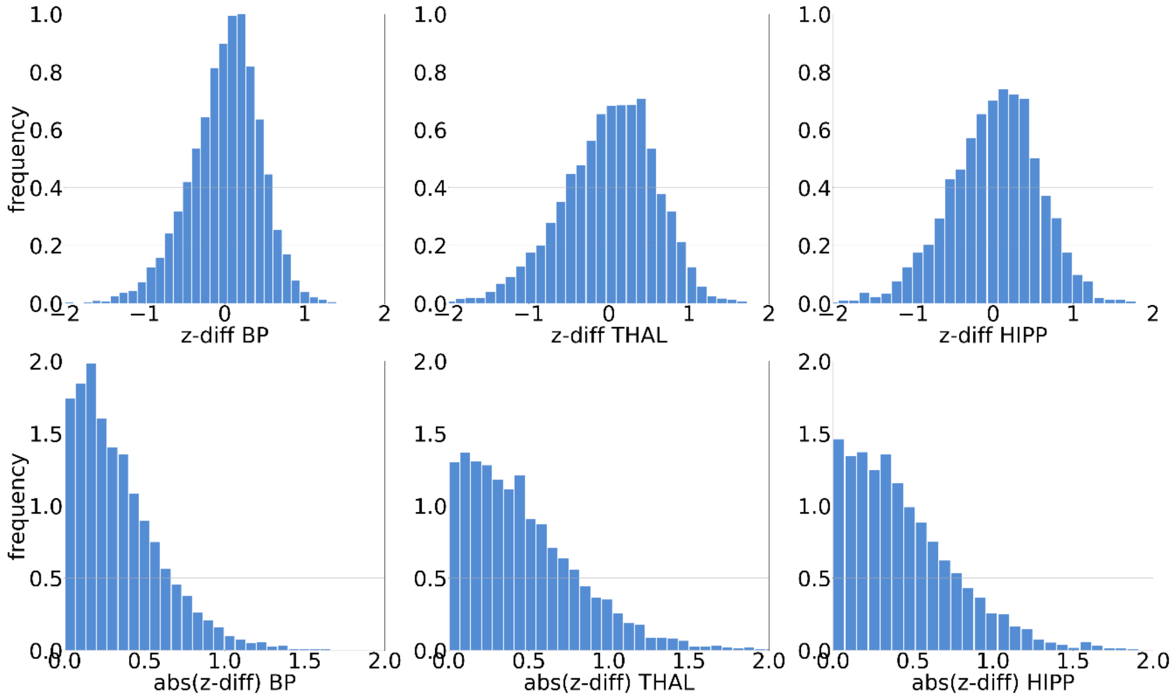

Supplementary Fig. 3 Histograms of z-diff (top) and the absolute value of z-diff (bottom) in the normal database (left: BP, middle:THAL, right: HIPP)

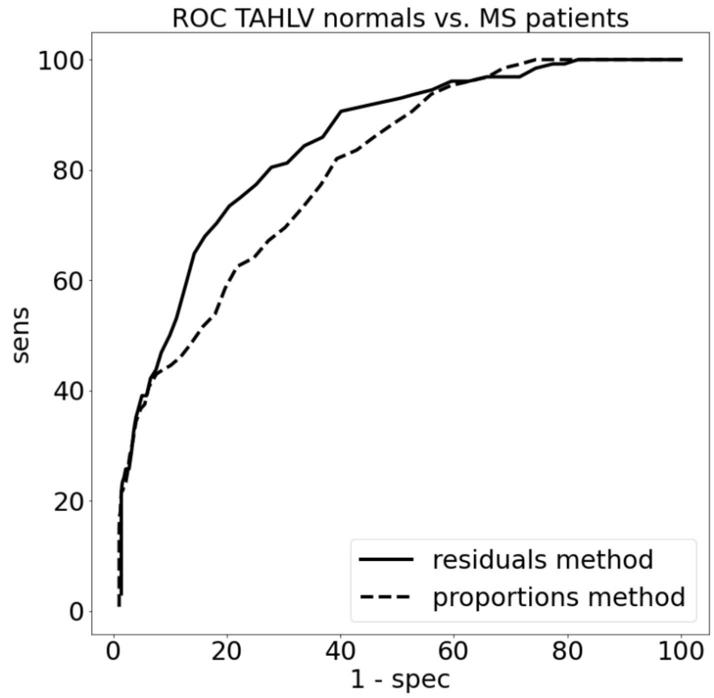

Supplementary Fig. 4 ROC curves of z-resTHALV (z-score of the TIV- and age-adjusted thalamus volume with the residuals method) and z-resTHALF (z-score of the age-adjusted thalamus-to-TIV ratio with the proportions method) for the discrimination of MS patients from the control subjects in the normal database

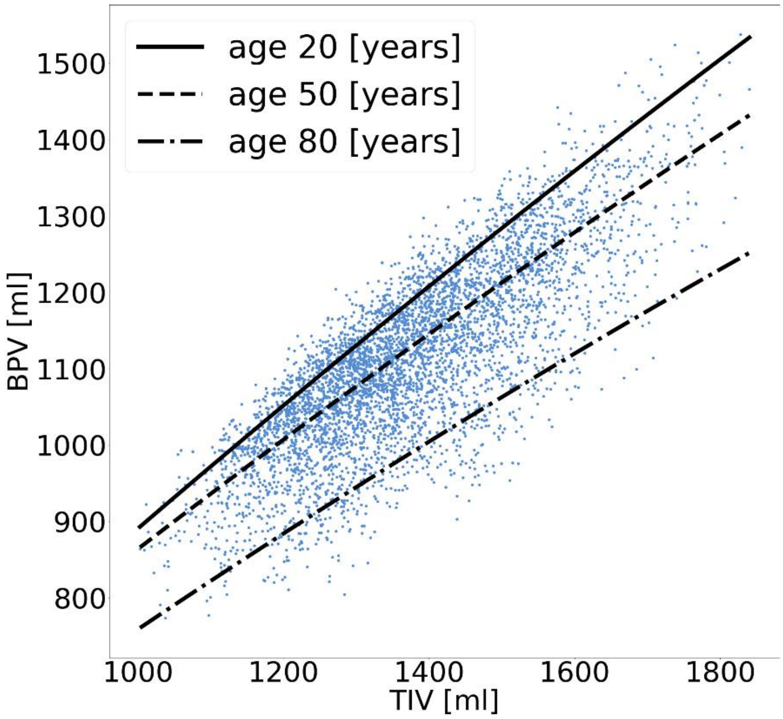

Supplementary Fig. 5 Final regression model in the residuals method evaluated at age 20, 50, and 80. The resulting curves are plotted together with the control subjects in the normal database
